# Prevalence of sleep-related problems and risks in a community-dwelling older adult population: a cross-sectional survey-based study

**DOI:** 10.1186/s12889-022-14443-8

**Published:** 2022-11-08

**Authors:** Nancy P. Gordon, Jimmy H. Yao, Leslea A. Brickner, Joan C. Lo

**Affiliations:** 1 Division of Research, Kaiser Permanente Northern California, 2000 Broadway, Oakland, CA 94612 USA; 2 The Permanente Medical Group, Oakland, CA USA; 3 Department of Adult and Family Medicine, Kaiser Permanente Oakland Medical Center, CA Oakland, USA

**Keywords:** Sleep, Sleep health, Sleep problems, Insomnia, Daytime fatigue, Older adults, Sleep aids, Lifestyle medicine, Physician advice

## Abstract

**Background:**

Despite evidence of adverse health consequences of inadequate restorative sleep for older adults, assessment of sleep quantity, quality, and use of sleep aids is not routinely done. We aimed to characterize sleep problems, sleep risks, and advice received about sleep in a community-dwelling older adult population, overall and in subgroups with health conditions and functional difficulties.

**Methods:**

This cross-sectional study used weighted self-report data for 5074 Kaiser Permanente Northern California members aged 65-79y who responded to a 2017 or 2020 Member Health Survey. We estimated usual amount of sleep (< 6, 6 to < 7, ≥7 hours) and prevalence of sleep problems (frequent insomnia, frequent daytime fatigue, poor quality sleep, and potential sleep apnea (OSA) symptoms (frequent very loud snoring, apnea episodes)) for older adults overall, by self-rated health, and in subgroups reporting hypertension, diabetes, heart disease, frequent problems with balance/walking, and frequent memory problems. We also estimated percentages who regularly used sleep aids and had discussed sleep adequacy with a healthcare professional in the past year.

**Results:**

Approximately 30% of older adults usually got less than the recommended ≥7 hours sleep per day, and 9% experienced frequent daytime fatigue, 13% frequent insomnia, 18% frequent insomnia/poor quality sleep, and 8% potential OSA symptoms. Prevalence of frequent insomnia was higher among women than men (16% vs. 11%). Higher percentages of those in fair/poor health and those with frequent balance/walking and memory problems reported sleeping < 6 hours per day and having all four types of sleep problems. Nearly 20% of all older adults (22% of women vs. 17% of men) and 45% of those with frequent insomnia (no sex difference) reported regular sleep aid use. Only 10% of older adults reported discussing sleep with a healthcare professional whereas > 20% reported discussing diet and exercise.

**Conclusions:**

Large percentages of older adults experience sleep problems or get less sleep than recommended for optimal sleep health. Older patients should routinely be assessed on multiple components of sleep health (sleep hygiene, quantity, quality, problems, and sleep aid use) and educated about sleep hygiene and the importance of getting adequate restorative sleep for their overall health and wellbeing.

## Introduction

Getting a sufficient amount of good quality sleep is increasingly recognized as an important contributing factor for primary and secondary prevention of metabolic conditions such as obesity, diabetes, hypertension, and coronary heart disease; for accidents such as falls, occupational injuries, and motor vehicle and other transportation accidents; for medical and industrial errors and accidents; and for optimal health and well-being [1–7]. Short sleep duration (generally defined as < 6 hours of sleep in a 24-hour period) and poor-quality sleep have also been associated with increased risk of neurocognitive disorders, including mild cognitive impairment and Alzheimer’s disease [8–11]. Depression and anxiety have a bidirectional relationship with inadequate or impaired sleep, especially insomnia. There is some evidence that suboptimal sleep factors, such as insomnia and inadequate sleep, may contribute to the onset of these mood disorders, and more extensive evidence that sleep issues can exacerbate existing depression and anxiety [12]. Depression and anxiety are also causal factors for inadequate sleep and/or poor sleep quality. Inadequate and poor-quality sleep, insomnia, sleep disordered breathing, and resulting excessive daytime sleepiness can also lead to problems with memory and cognition [13–15], gait speed and balance confidence [16], and increased risk of falls [17–19], as well as reduced quality of life [20, 21].

Short sleep duration, poorer sleep quality and efficiency, and sleep problems, including insomnia, fragmented sleep, sleep disordered breathing, and excessive daytime sleepiness, are more common among older than middle-aged and younger adults [22, 23]. The National Sleep Foundation (NSF) and American Academy of Sleep Medicine (AASM) recommend that all adults get at least 7 hours of sleep per day [24, 25], but a 2017 U.S. national survey suggests that 25% of adults aged 65 and older get less than that amount [26]. Other surveys suggest that one-third to two-thirds of adults in this older age group suffer from symptoms of insomnia [27–29] and sleep disordered breathing [30]. Despite the adverse health consequences of inadequate restorative sleep, sleep problems frequently are undiagnosed and untreated, particularly in older adults [4, 31]. Indeed, a recent national survey found that over half of older adults thought that poor sleep was a normal part of aging [32]. While increasing sleep difficulty may result from normal changes in “sleep architecture” [23, 33], chronic health issues, and/or medication side effects, older adults and their healthcare providers should not simply accept sleep problems as a normal part of aging without attempting to remediate them. A recent study found that adults with sleep disorders such as insomnia and obstructive sleep apnea (OSA) have significantly higher rates of health care utilization and health care expenditures, suggesting that screening and intervention for sleep-related problems could be an important component of controlling health care costs [34].

In this study, we used member health survey data to estimate the percentages of adults aged 65-79 years in a community-dwelling population who get less than the recommended amount of sleep per day, who do not try to get enough sleep, who have sleep problems, and who regularly use sleep medicines and supplements, with special focus on subgroups with cardiovascular risks and conditions, functional difficulties, and fair/poor health status which may be exacerbated by inadequate or poor quality sleep. We also examined whether older adult patients were as likely to recall receiving advice about getting enough sleep as compared to advice about diet and exercise. Our goal was to provide more information about the burden of sleep issues among community-dwelling older patients that can be used to promote change related to the assessment and treatment of sleep issues in primary care settings.

## Methods

### Data source

For this cross-sectional study, we used data for 5074 Kaiser Permanente Northern California (KPNC) members aged 65-79 years who responded to the 2017 or 2020 KPNC Member Health Surveys (MHS). KPNC is an integrated healthcare delivery system serving over 4 million adults in a region covering the greater San Francisco Bay Area and Sacramento and Central Valley areas. Most adults aged 65-79 are covered by KPNC’s Medicare Advantage plan, which includes an annual visit during which primary care providers are expected to review health risk behaviors and provide advice to help their older patients maintain or improve their health. The MHS uses a self-administered (print or online) questionnaire to collect data from an age-gender stratified random sample of current KPNC health plan members ages 25-90 years who were members in last quarter of the year preceding the survey. The response rate for the combined MHS2017 and MHS2020 cycles for adults aged 65-79 was 50.8% (*N* = 5074/9979). More information about the MHS is available in an earlier publication [35] and on the survey website (www.memberhealthsurvey.kaiser.org). Additional descriptive data about sociodemographic and health behavior characteristics of KPNC members in this age group based on other MHS cycles can be found in other publications [36, 37].

### Study variables

*Sleep characteristics*. Total amount of sleep in a 24-hour period was derived from numeric response to the question “On a typical weekday, how many total hours of sleep do you usually get, including naps?” Responses were rounded to the nearest half hour and categorized as < 6 hours, 6 to < 7 hours, and ≥ 7 hours per day (the number of recommended sleep hours for adults in this age group). While < 7 hours is considered to be insufficient sleep for most people, < 6 hours has become the generally accepted definition of “short sleep” [38]. Frequent insomnia was based on a single checklist item “frequent problems falling or staying asleep” during the past 12 months. Sleep quality was based on the question “How would you rate the usual quality of your sleep?” with possible responses of very good, good, fair, poor, or very poor. For our analyses, we combined “poor” and “very poor” responses into “poor-quality” sleep. Approximately 33% of those with frequent insomnia reported poor-quality sleep and 49% of those with poor-quality sleep reported frequent insomnia. Since we considered frequent insomnia to be an aspect of poor-quality sleep, we combined these indicators into a variable “frequent insomnia and/or poor-quality sleep.” Frequent daytime fatigue was identified by the checklist item “frequently felt very sleepy/tired during the time of day you normally work or do other daily activities.” Potential obstructive sleep apnea (OSA) symptoms was defined by a positive response to at least one of two health checklist items asking whether they frequently snored very loudly and whether they sometimes stopped breathing in their sleep or woke up feeling like they were choking or gasping for air. A separate health history checklist item asked whether they ever had “sleep apnea (OSA).” Use of sleep aids during the past 12 months was derived from two checklist items that asked about use of prescription or non-prescription sleep medicine at least twice a week and use of melatonin or a sleep formula containing melatonin. Intentional sleep behavior (“try to get enough sleep to feel well rested”) came from a checklist question which asked about behaviors individuals were engaging in to improve or maintain their health.

*Advice received about getting enough sleep* was based on response to a checklist question about whether the individual had talked with or received recommendations from a doctor or other healthcare professional about several different health-related behaviors in the past 12 months. Receipt of advice about exercise and diet were from the same checklist.

*Demographic and health condition* s*ubgroups* included 5-year age group, self-reported sex, and health status (excellent/very good, good, or fair/poor). Cardiovascular risks/conditions were assigned based on responses to a question that asked whether the individual had had, was treated for, or used medication or a special diet for a list of health conditions and to a question that asked about use of a list of prescription and over-the-counter medications at least twice a week during the prior 12 months. People were considered to have diabetes if they had or were being treated for diabetes (*other than only during pregnancy)* or were taking insulin or other diabetes medicine; hypertension was assigned based on indication of high blood pressure *(diagnosed by a clinician*) in the past 12 months or regular use of high blood pressure medicine; heart disease was assigned if they indicated having heart disease *(*e.g.*, heart attack, angina, blocked artery, atrial fibrillation, congestive heart failure)* in the past 12 months, or ever had heart disease, heart surgery, or a heart attack, or if they were taking heart medicine). The same health conditions checklist was used to identify older adults with frequent problems with balance/walking and frequent problems with memory.

### Statistical analysis

All analyses were performed with survey data weighted to the age-sex-racial composition of the KPNC adult membership in 2019 using SAS v9.4 (SAS Institute, Cary, NC) Proc Surveyfreq and Proc Surveymeans [39]. For the sleep-related outcomes, we produced prevalence estimates with 95% confidence intervals (CIs) for the overall population and for demographic and health status subgroups. We also estimated percentages of older adults using sleep aids (prescription or over-the-counter medications and melatonin) and getting advice from their healthcare team about sleep and other health behaviors. All estimates reported are based on weighted survey data. We assessed differences by sex and age group using chi-square tests, with a *p*-value of < 0.05 used to determine statistical significance. We used modified log-Poisson regression models to estimate the adjusted prevalence ratio (aPR) of sleep characteristics when comparing levels of health status after controlling for age as a continuous variable, sex, and race/ethnicity. All confidence intervals are presented at the 95% confidence limit, and between-group differences cited in the text are statistically significant at *p < 0.05.*

## Results

### Cohort characteristics

Among 5074 older adults, the weighted analytic sample was 54.5% female, with 41.9% aged 65-69, 35.3% aged 70-74, and 22.8% aged 75-79. Two-thirds (66.2%) were non-Hispanic White, 7.4% Black, 8.8% Hispanic, 15.9% Asian/Pacific Islander, and 1.6% other race, and 66% were married/living as married, with men more likely than women (78% vs. 57%) to be living with a partner. Approximately 62% had at least 1 of 3 cardiovascular risks or conditions (18.7% diabetes, 53.9% hypertension, 16.7% heart disease), 9.5% had frequent problems with balance or walking, and 6.0% experienced frequent problems with memory. Most older adults considered their health to be good (40.5%) or very good/excellent (45.8%), with only 15.1% rating their health as fair or poor.

### Sleep quality

**Fig. 1:**
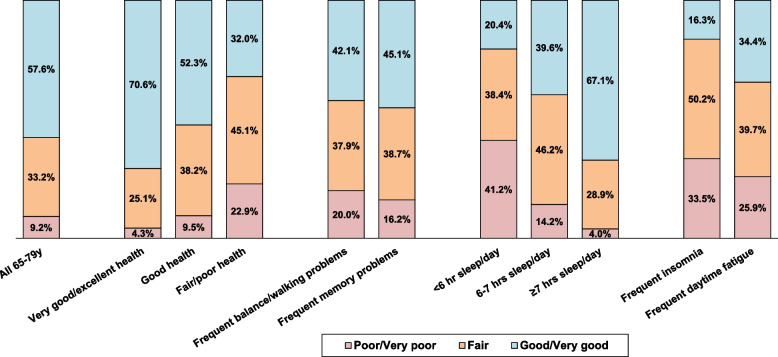
Rating of overall sleep quality, by health status and presence of sleep problems

Estimated percentages of older adults who rate their sleep quality as good or very good, fair, or poor are shown in Fig. 1. Overall, approximately 58% of older adults rated their sleep quality as good or very good, 33% as fair, and 9% as poor, with no significant differences by sex or age group. The distribution of sleep quality rating among those with the cardiovascular risks/conditions was very similar to the overall population. Older adults with very good/excellent health and those who got ≥7 hours of sleep per day were more likely to report good sleep quality and less likely to report poor sleep quality than the overall population. However, higher proportions of older adults with frequent memory problems, frequent problems with balance/walking, fair/poor health, frequent insomnia, frequent daytime fatigue, and those who got < 6 hours or 6 to 7 hours of sleep per day rated their sleep quality as poor (and lower proportions rated their sleep quality as good or very good) than the overall population.

### Sleep quantity and sleep problems

Overall, 28.2% (CI: 26.8-29.5%) of older adults usually got < 7 hours and 7.7% (CI: 7.0-8.5%) got < 6 hours sleep per day (Table 1). Sleep quantity did not significantly differ by sex, but controlling for sex, adults aged 65-69 were less likely than those aged ≥70 years to get at least 7 hours of sleep per day. Frequent daytime fatigue was experienced by 9% of older adults, with no significant difference by sex, and was a less common problem for adults aged 70-74 than the younger and older age groups. Approximately 13% of older adults had frequent insomnia and 18.1% had frequent insomnia or poor-quality sleep, with these problems more prevalent among women than men. Potential OSA symptoms were experienced by 8% of older adults during the past 12 months, with men more likely to report ≥1 of these symptoms than women. Additionally, approximately 16% (15.7%, CI: 14.7-16.8%) of older adults indicated ever having OSA, including a higher percentage among men (20.1%) than women (12.1%). Approximately 73% (66.0-79.0%) of older adults who reported previous apnea episodes and 47% (41.1-53.6%) who frequently snored very loudly had been told in the past that they had OSA.

**Table 1:** Sleep quantity and quality by sex, age group, and health attributes

Subgroup		Quantity of Sleep (total hours per day, including naps)			Frequent daytime fatigue	Frequent insomnia	Frequent insomnia or poor sleep quality	Frequent very loud snoring and/or apnea episodes ^a^
		< 6 hours	6 to < 7 hours	≥7 hours				
	N ^**b**^	% (95% CI)	%	% (95% CI)	% (95% CI)	% (95% CI)	% (95% CI)	% (95% CI)
All	5074	7.7 (7.0-8.5)	20.4	71.8 (70.5-73.1)	9.0 (8.2- 9.8)	13.4 (12.4-14.4)	18.1 (16.9-19.2)	8.0 (7.2-8.8)
**Sex**								
Men	2455	7.3 (6.2-8.4)	19.8	72.9 (71.0-74.7)	9.7 (8.5-11.0)	10.8 (9.5-12.1)	15.0 (13.5-16.5)	9.7 (8.4-10.9)
Women	2619	8.1 (7.0-9.1)	21.0	71.0 (69.1-72.8)	8.4 (7.3- 9.5)	15.6 (14.1-17.1) ^c^	20.6 (19.0-22.3) ^c^	6.6 (5.6- 7.7) ^c^
**Age**								
65-69 years	1640	8.1 (6.8-9.4)	22.3	69.6 (67.3-71.8)	9.7 (8.2 – 11.1)	13.5 (11.9-15.2)	18.6 (16.7-20.5)	9.1 (7.7-10.5)
70-74 years	1602	6.9 (5.7-8.2)	19.9	73.2 (71.0-75.4)	7.2 (5.9 – 8.4)	12.7 (11.0-14.3)	16.9 (15.0-18.8)	8.2 (6.8- 9.5)
75-79 years	1832	8.4 (7.1-9.6)	17.7	73.9 (71.8-76.0)	10.6 (9.1-12.1)	14.4 (12.7-16.0)	18.9 (17.0-20.7)	5.9 (4.8- 7.0) ^d^
**Cardiovascular risks/conditions**								
Hypertension	2893	8.3 (7.2-9.3)	21.5	70.2 (68.5-72.0)	10.5 (9.4-11.7)	14.3 (12.9-15.7)	19.9 (18.4-21.5)	10.5 (9.3-11.7)
Diabetes	1047	9.4 (7.6-11.2)	23.0	67.6 (64.5-70.6)	12.8 (10.6-14.9)	14.0 (11.7-16.2)	19.9 (17.3-22.5)	11.0 (8.9-13.0)
Heart Disease	914	8.1 (6.2-9.9)	19.8	72.1 (69.0-75.3)	14.5 (12.1-16.9)	15.9 (13.4-18.5)	21.4 (18.6-24.3)	10.9 (8.8-13.1)
**Functional difficulties**								
Frequent problems with balance/walking	531	12.5 (9.5-15.6)	19.9	67.6 (63.2-71.9)	31.1 (26.8-35.3)	31.5 (27.2-35.8)	38.5 (34.1-43.0)	20.9 (17.1-24.7)
Frequent problems with memory	335	12.6 (8.9-16.3)	20.4	67.0 (61.6-72.4)	33.3 (27.8-38.7)	35.6 (30.1-41.2)	39.9 (34.3-45.6)	23.7 (18.7-28.7)
**Overall health status**								
Very good/ Excellent	2208	5.1 (4.1- 6.0)	19.5	75.4 (73.5-77.4)	3.9 (3.0- 4.7)	9.3 (8.0-10.6)	11.7 (10.2-13.1)	5.5 (4.5- 6.5)
Good	2032	8.9 (7.6-10.2)^e^	20.4	70.7 (68.6-72.8) ^e^	9.9 (8.5-11.3) ^e^	14.2 (12.6-15.8) ^e^	18.7 (16.9-20.5) ^e^	8.4 (7.1- 9.7) ^e^
Fair/Poor	819	12.9 (10.5-15.3)^e, f^	23.2	63.9 (60.3-67.5) ^e, f^	22.3 (19.3-25.4) ^e, f^	23.9 (20.8-27.1) ^e, f^	35.5 (32.0-39.0) ^e, f^	14.6 (12.0-17.2) ^e, f^

^a^ Symptoms indicative of potential obstructive sleep apnea

^b^ Subgroup Ns for some sleep risk indicators are smaller than those shown due to missing data

^c^ Significant (*p* < .05) sex difference

^d^ Significant (*p* < .05) age group difference

^e^ Significantly (*p* < .05) different from very good/excellent health after adjusting for age and sex

^f^ Significantly (*p* < .05) different from good health after adjusting for age and sex

Among older adults with diabetes, hypertension, and/or history of heart disease, approximately 30% usually got < 7 hours and 8-9% < 6 hours of sleep per day. They were similar to the overall population with regard to prevalence of frequent insomnia or frequent insomnia/poor quality sleep, but were more likely to report frequent daytime fatigue, potential OSA symptoms, and ever having OSA than the overall population. Among older adults with frequent balance/walking problems and frequent memory problems, approximately one-third got < 7 hours and 12% got < 6 hours of sleep daily. Additionally, 31-33% had frequent daytime fatigue, 32-36% had frequent insomnia, 39% frequent insomnia/poor quality sleep, and approximately 20% experienced OSA symptoms during the previous 12 months.

**Fig. 2:**
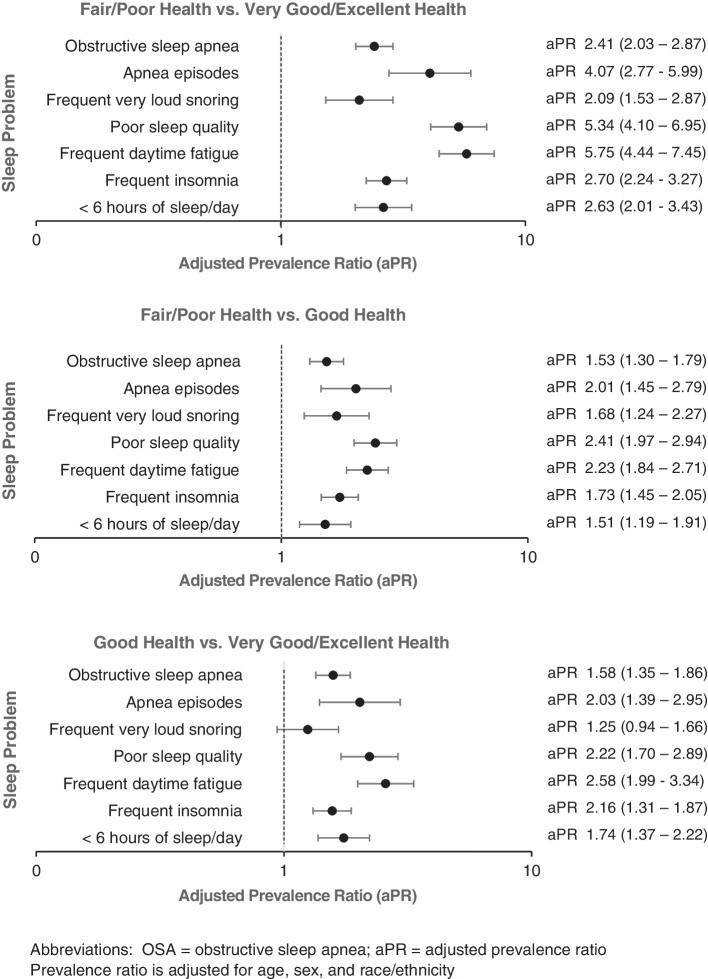
Comparison of prevalence of sleep problems in older adults by self-rated health status

Older adults who rated their health as fair or poor were more likely to have sleep problems/risks than those reporting good or very good/excellent health, and those who reported good health were more likely to have most of the examined sleep problems/risks than those who rated their health as very good or excellent. In addition to sleep issues shown in Table 1, older adults with fair/poor health were more likely than those with good or very good/excellent health to indicate having poor sleep quality and having been told that they have OSA (26.9% vs. 17.2 and 10.9%, respectively). These differences in sleep problems/risks across health status categories remained statistically significant after controlling for age, sex, and race/ethnicity (Fig. 2).

### Overlap of sleep problems

**Fig. 3:**
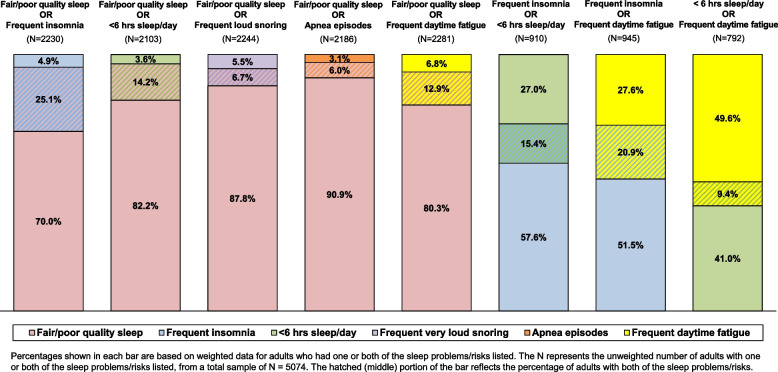
Extent to which sleep risks and problems overlap in an older adult population

Among older adults who got < 6 hours of sleep per day, 81.2% (CI: 77.2-85.2%) also had frequent insomnia or fair/poor quality sleep. Figure 3 illustrates the extent to which sleep problems overlap and the implications of non-overlap for potential missed identification of the other problem based on pairing risks and then restricting analyses to those with one or both risks (e.g., fair/poor sleep and/or frequent insomnia). Asking only about fair/poor quality sleep would potentially miss approximately 16% of older adults with frequent insomnia, 20% who get < 6 hours sleep per day, 35% with frequent daytime fatigue, 45% with frequent loud snoring, and 34% with apnea episodes. Asking only about frequent insomnia would potentially miss 74% of older adults with fair/poor sleep quality, 71% with frequent daytime fatigue, and 64% who usually get < 6 hours of sleep per day. Identifying only those who usually get < 6 hours of sleep daily would potentially miss 85% of older adults with fair/poor quality sleep, 79% with frequent insomnia, and 81% with frequent daytime fatigue. Finally, asking only about frequent daytime fatigue would potentially miss 84% of those who get < 6 hours sleep per day. We also examined the relationship between usual number of hours of sleep and experience of frequent daytime fatigue, finding a U-shaped association. Specifically, frequent daytime fatigue was indicated by about 19% of older adults who usually got < 6 hours of sleep, 9% who got 6 to < 7 hours, 7% of those who got 7 to 9 hours, and 20% of those who got ≥9.5 hours.

### Intentional sleep behavior

Approximately 75% (CI: 74.1-76.6%) of older adults indicated that they try to get enough sleep to feel well-rested, with women more likely than men to make this effort (77.6% vs. 72.6%). Older adults who indicated getting < 6 hours of sleep per day were less likely to try to get enough sleep than those who got 6 to < 7 hours and ≥ 7 hours of sleep per day (58.1% vs. 69.4 and 79.3%, respectively), and older adults with fair or poor sleep quality were less likely to try to get enough sleep than those with good sleep quality (62.3% vs. 76.7%).

### Regular use of sleep aids

Overall, 19.9% (CI: 18.7-21.1%) of older adults used a sleep aid at least twice a week, with 12.7% (CI: 11.7-13.7%) using a prescription or over-the-counter (OTC) sleep medication and 12.6% (CI: 11.6-13.6%) using melatonin. Women were more likely than men to use a sleep aid (22.4% vs. 16.9%), a prescription or OTC sleep medication (14.4% vs. 10.5%), and melatonin (14.1% vs. 10.9%). Among older adults who had frequent insomnia, 45.3% (CI: 41.3-49.2%) reported regularly using a sleep aid, with 33.6% (CI: 29.8-37.4%) using a prescription or OTC sleep medication and 27.6% (24.0-31.3%) using melatonin, with no observed sex difference.

### Receipt of advice from healthcare professionals about getting enough sleep

**Fig. 4:**
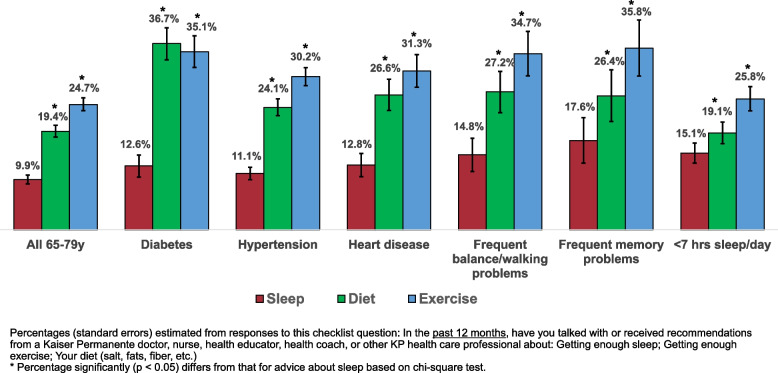
Percentages of older adults who reported discussing or receiving advice about adequate sleep, diet, and exercise

Approximately 10% (9.9%, CI: 9.0-10.8%) of older adults reported receiving advice to get enough sleep from a healthcare professional during the prior year, less than half the percentage who reported receiving advice about their diet (19.4%, CI: 18.2-20.5%) and getting enough exercise (24.6%, CI: 23.4-25.5%) (Fig. 4). Among those with ≥1 of the chronic cardiovascular risks/conditions, only 11-12% recalled advice about getting enough sleep compared to approximately 30-35% who received advice about exercise and 24-36% who received advice about their diet. Only 14.8% of older adults with frequent balance/walking problems and 17.6% of those with frequent memory problems received advice to get enough sleep. While older adults with sleep issues that could cause a sleep deficit (< 6 hours of sleep daily, frequent insomnia, and frequent daytime fatigue) were twice as likely (21-23%) as the overall population to recall receiving advice about getting enough sleep, the majority of those with these sleep issues did not recall receiving such advice. Men who had received advice about getting enough sleep were more likely than those who did not to indicate that they tried to get enough sleep (79.4% vs. 71.9%) but getting advice did not appear to affect sleep intentions among women.

## Discussion

In this survey of community-dwelling older adults aged 65-79 who received care in the same Northern California healthcare delivery system, we found that over one-fourth got less than the 7 hours of sleep recommended for older adults by the NSF and AASM and over 40% were burdened with fair/poor sleep quality, 13% with frequent insomnia, and 9% with frequent daytime fatigue and symptoms indicative of possible sleep apnea. Consistent with other studies that identified a higher prevalence of sleep problems among older adults with fair or poor health or self-reported limitations in physical functioning [32, 40], our study also found a higher prevalence of insomnia, poor quality sleep, and frequent very loud snoring or apnea episodes among older adults with fair/poor health, frequent balance/walking problems, and frequent memory problems. We also found that nearly 20% of older adults and 45% of those with frequent insomnia regularly used a sleep aid, estimates in line with those found by a 2017 U.S. national survey [32]. Older adults with balance or gait problems who also experience frequent insomnia or inadequate or fragmented sleep may be at higher risk for falls, especially at night if they are using pharmacological sleep aids [41, 42].

We observed a higher prevalence of frequent insomnia and higher prevalence of use of sleep aids among women and higher prevalence of frequent very loud snoring and/or apnea episodes among men. These sex differences, which have been documented in other studies [43–45], have ramifications for screening for and diagnosis of sleep problems in the primary care setting. Specifically, fragmented sleep due to sleep problems like insomnia, OSA, and nocturia, can result in subjective complaints such as daytime fatigue or sleepiness, exhaustion, difficulty concentrating, and memory problems, all of which overlap with symptoms of psychological conditions such as anxiety and depression. It has been suggested that because of the extensive overlap of symptoms of depression with symptoms of insomnia and fragmented sleep and because women are less likely to report loud snoring and apnea episodes (elements of standard OSA screening tools), many women with sleep problems are not being referred by their primary care provider to a sleep specialist for more thorough clinical assessment and may even be misdiagnosed and treated for depression instead of the underlying sleep problem [46, 47]. Studies have found that addressing sleep problems in patients with depression can lessen depressive symptoms and improve cognitive functioning and quality of life [12].

In his seminal 2014 article, Buysse called for the field of sleep to move beyond a strictly medical model of screening for and treating sleep problems based on clinical diagnosis thresholds to embracing a more preventive medicine/public health framework of sleep health. Buysse defined sleep health as “a multidimensional pattern of sleep-wakefulness, adapted to individual, social, and environmental demands, that promotes physical and mental well-being”, with good sleep health “characterized by subjective satisfaction, appropriate timing, adequate duration, high efficiency, and sustained alertness during waking hours” [48]. Since then, the concept of sleep health has been taken up by medical and public health organizations. The American Academy of Sleep Medicine and the Sleep Research Society have called for paying greater attention to sleep health in clinician and patient education, the continuum of clinical care, occupational and worksite health, health promotion, and research [49]. The American College of Lifestyle Medicine considers restorative sleep to be one of the six pillars of lifestyle medicine [6], and the American Heart Association recently added sleep health to its cardiovascular health checklist Life’s Essential 8™ [50]. The U.S. Office of Disease Prevention and Health Promotion’s (ODPHP) *Healthy People 2020* and *Healthy People 2030* reports include a goal of increasing public knowledge of how adequate sleep and treatment of sleep disorders improve health and safety, productivity, and quality of life with stated objectives to increase the percentages of adults who get ≥7 hours of sleep per day and have sleep apnea symptoms evaluated by a healthcare provider [26].

Despite the growing awareness in the medical community regarding the importance of healthy sleep, in our study, we found that older adults overall and in subgroups with chronic health problems were less likely to recall being advised by a healthcare professional about getting enough sleep than about getting enough exercise or about their diet. This finding is in line with other studies that suggest that primary care providers are underscreening their patients for sleep problems [51, 52] and that the majority of patients are not reporting symptoms indicative of sleep problems to their primary care providers [53–56]. There is a growing call for sleep health to be assessed as a behavioral vital sign [57–59] not only in adult medicine settings, but also in mental health settings based on evidence that sleep disorders and inadequate sleep not only contribute to poor cardiovascular health and functional difficulties, but also increase development and exacerbation of cognitive and mental health problems [60]. There is a growing body of evidence that non-pharmacological interventions like cognitive behavioral therapy, mindfulness meditation, and sleep hygiene counseling can positively impact sleep problems in older adults [61, 62]. In addition, reducing loud snoring, apnea episodes, or the tossing and turning related to insomnia in one sleep partner can improve sleep health of the other partner, resulting in improved physical and mental health of both partners and the well-being of their relationship.

The challenge in real world clinical settings is the limited time available to screen for sleep problems experienced by older adult patients as part of a comprehensive behavioral vital sign assessment at clinic visits or multi-domain health risk assessment questionnaire. Future research is needed to develop a concise and practical set of questions that can be used in the clinic setting to characterize overall sleep health and to identify patients in need of further assessment or sleep hygiene education for specific problems. Our study also suggests that asking only one question about overall sleep quality or usual number of hours of sleep, but not questions about daytime fatigue, insomnia, snoring, and sleep disordered breathing may not uncover sleep health issues that many patients don’t report to their primary care provider.

Insomnia is one of the most common and debilitating sleep complaints among older adults, with an estimated one-third to one-half of this age group experiencing trouble falling or staying asleep [22]. One novel way of screening for insomnia may be using the “Trouble falling or staying asleep, or sleeping too much” item on the Patient Health Questionnaire (PHQ-9) depression screener [63]. Several studies have found that this item is highly correlated with score on the Insomnia Severity Index [64–66]. This question could potentially be added to the widely used PHQ-2 screener, but treated as a separate insomnia risk domain rather than as a component of a depression risk score; used as a standalone indicator of insomnia in health risk screeners; or added to other brief screeners for sleep problems such as the STOP-BANG for obstructive sleep apnea, which already captures daytime fatigue, snoring, and apnea episodes [67].

Our study has several strengths. Our estimates are based on a large and sociodemographically diverse respondent sample of older adults who received health care in the same integrated healthcare delivery system, including large subgroups of adults with cardiometabolic and functional health conditions that research suggests are affected by amount and quality of sleep. The survey was conducted with a stratified random sample of health plan members that was weighted to reflect the age-sex-racial distribution of the health plan membership. We examined multiple indicators of sleep health, including amount of usual amount and quality of sleep, frequent insomnia, very loud snoring and apnea episodes, and use of pharmacological sleep aids. Finally, we were able to compare proportions of adults who recalled receiving advice during the prior year from health plan healthcare professionals about getting enough sleep versus about their diet and getting enough exercise.

We acknowledge that our study has limitations. Our estimates of sleep health variables were based on single-item self-reported measures, not validated physiological measurements and longer validated screening tools for sleep quality, insomnia, and obstructive sleep apnea. However, these types of single-item questions are more likely to be adopted for first-stage screening in the outpatient clinic setting or health risk assessment where time or space on a questionnaire precludes using lengthier screening tools. Due to limitations of our study cohort, we did not examine racial/ethnic group differences in sleep health in this older adult population. There is an extensive body of research showing racial/ethnic differences in sleep patterns and sleep health [68], and this should continue to be an important focus of future research as poor sleep health may be a causal factor in racial/ethnic health disparities [69]. Our survey sample was restricted to adults whose primary language was English, limiting generalizability to English speakers. Finally, because the survey sample came from one Northern California health plan, the results from this study may not reflect the populations of older adults in other health plans or other regions in the U.S., nor adults who are uninsured.

## Conclusion

Large percentages of older adults experience sleep problems or do not get the number of hours of good quality sleep recommended for optimal sleep health. Primary care and mental health providers should more routinely ask their older patients about multiple components of sleep health (i.e., sleep hygiene, sleep quantity, sleep quality, sleep problems, and use of sleep aids). As part of a lifestyle medicine approach to healthcare, providers should routinely educate their older patients about the importance of healthy sleep for prevention and management of physical, functional, mental, and cognitive health problems and counsel them about how to optimize their sleep health. Finally, when sleep problems are identified, providers should conduct or arrange for an in-depth assessment of factors contributing to the sleep problem so that appropriate treatment or sleep hygiene counseling can be offered.

## Data Availability

The Kaiser Permanente Northern California (KPNC) Institutional Review Board has not provided approval for Member Health Survey data to be placed in a public access repository. However, researchers can request access to use this study data by contacting the corresponding author (NG) or the DOR Data Sharing Workgroup at DOR-DataSharingWorkgroup@kp.org.

## Abbreviations

AASM: American Academy of Sleep Medicine
APR: Adjusted prevalence ratio
MHS: Member Health Survey
KPNC: Kaiser Permanente Northern California
NSF: National Sleep Foundation
ODPHP: Office of Disease Prevention and Health Promotion
OSA: Obstructive sleep apnea
OTC: Over-the-counter
US: United States
